# Myxoedema Coma With Type 2 Respiratory Failure and Hypothermia in an Oedematous Patient: A Case Report

**DOI:** 10.7759/cureus.116357

**Published:** 2026-09-16

**Authors:** Basit Khan, Amal Qaisar, Olivia Owen, Wael Gadalla

**Affiliations:** 1 Renal Medicine, University Hospitals of Leicester (UHL) NHS Trust, Leicester, GBR; 2 Nephrology, University Hospitals of Leicester (UHL) NHS Trust, Leicester, GBR; 3 Intensive Care Unit, University Hospitals of Leicester (UHL) NHS Trust, Leicester, GBR

**Keywords:** cold hypercapnia, heart failure, hypothyroidism, hypothyroid myxedema coma, type 2 respiratory failure

## Abstract

Myxoedema coma is a rare, life-threatening endocrine emergency representing the most severe form of hypothyroidism. We present the case of a South Asian woman in her early 70s admitted with generalised oedema, chest pain, general malaise, and reduced appetite. Initially treated as decompensated heart failure, she deteriorated with hypothermia, hypotension, bradypnea, and reduced consciousness, requiring intensive care admission and intubation. Thyroid function testing revealed profound hypothyroidism (TSH 67 mU/L, T4 <2 pmol/L), confirming myxoedema coma. She received intravenous liothyronine and levothyroxine with clinical improvement. This case highlights the noteworthy phenomenon of cold hypercapnia alongside highlighting the diagnostic challenges of myxoedema coma, which may mimic or exacerbate cardiac and respiratory failure. Clinicians should maintain a high index of suspicion in at-risk patients, as delayed recognition and treatment significantly increase mortality.

## Introduction

Myxoedema coma is a rare, life-threatening endocrine emergency representing the most severe manifestation of hypothyroidism. Despite advances in intensive care management and thyroid hormone replacement therapy, it remains associated with substantial mortality, with reported mortality rates ranging from 25% to 60% [1-3]. Although the term “myxoedema coma” is widely used, many patients do not present in a state of coma. Instead, clinical manifestations exist on a spectrum ranging from confusion and lethargy to severe encephalopathy and coma [1-3].

The condition predominantly affects older adults, particularly women with longstanding untreated or inadequately treated hypothyroidism [1-3]. This reflects both the increased prevalence of thyroid dysfunction among females and the higher frequency of hypothyroidism with advancing age [4,5]. In Europe, hypothyroidism is relatively common, with population-based studies reporting an overall prevalence of approximately 1-2%, although a proportion of cases remain undiagnosed [4,6]. Patients with previously unrecognised hypothyroidism are at risk of acute decompensation following physiological stressors, such as infection, cardiovascular events, trauma, surgery, exposure to cold temperatures, sedative medications, or respiratory compromise [1-3].

The pathophysiology of myxoedema coma is characterised by profound thyroid hormone deficiency, resulting in reduced cellular metabolism, impaired oxygen consumption, and decreased thermogenesis. These changes contribute to reduced myocardial contractility, decreased cardiac output, increased systemic vascular resistance, impaired renal perfusion, and reduced free-water clearance. Consequently, patients may develop fluid retention, hyponatraemia, pleural effusions, and pericardial effusions, which can mimic primary cardiac disease [7-9]. In addition, the accumulation of glycosaminoglycans in the interstitial tissues contributes to the characteristic non-pitting oedema of severe hypothyroidism [10].

Respiratory dysfunction is a recognised and potentially life-threatening complication of myxoedema coma. Reduced central respiratory drive, impaired ventilatory responses to hypoxia and hypercapnia, respiratory muscle weakness, and upper airway obstruction secondary to soft tissue oedema may result in hypoventilation and type 2 respiratory failure (characterised by a dangerous buildup of carbon dioxide) [11-13]. These manifestations can resemble more common clinical conditions, including chronic obstructive pulmonary disease, pneumonia, obesity hypoventilation syndrome, and decompensated heart failure, contributing to delays in diagnosis [12,14].

The diagnosis of myxoedema coma remains primarily clinical and should not be delayed while awaiting biochemical confirmation. Typical laboratory findings include markedly elevated thyroid-stimulating hormone (TSH) with profoundly reduced free thyroxine (FT4) concentrations in patients with primary hypothyroidism [1,3]. Diagnostic tools such as the scoring system proposed by Popoveniuc et al. may assist clinicians in recognising cases where the diagnosis is uncertain by incorporating thermoregulatory, neurological, cardiovascular, gastrointestinal, and metabolic features [15].

Management requires prompt thyroid hormone replacement alongside intensive supportive care, including ventilatory support when required, correction of metabolic abnormalities, treatment of precipitating factors, and empirical corticosteroid therapy when adrenal insufficiency cannot be excluded [1-3,16]. Early recognition and treatment are essential, as delays are associated with increased morbidity and mortality.

We present the case of a South Asian woman in her early 70s whose initial presentation was suggestive of acute decompensated heart failure before she deteriorated with hypothermia, hypercapnic respiratory failure, and reduced consciousness secondary to myxoedema coma. This case highlights the diagnostic challenges associated with severe hypothyroidism and describes the unusual physiological presentation of cold hypercapnia, where the metabolic effects of profound thyroid hormone deficiency appear to outweigh the expected vascular effects associated with hypercapnia.

## Case presentation

A South Asian woman in her early 70s presented to the Emergency Department with a five-day history of general malaise, poor appetite, bilateral ankle oedema, and reduced mobility. She also reported two days of non-radiating retrosternal chest pain. Her recent medical history included a previous intensive care admission with coronavirus disease 2019 (COVID-19). In the months preceding admission, she had attended her General Practitioner on several occasions with symptoms including fatigue, dry skin, and progressive hair thinning; however, thyroid dysfunction had not previously been investigated.

On initial assessment, she was hypothermic, with a temperature of 34.4°C. Her blood pressure was 190/97 mmHg, and her heart rate was 58 beats/minute. She was fully alert with a Glasgow Coma Scale (GCS) score of 15/15. Oxygen saturation was 96% while receiving 40% oxygen. Clinical examination demonstrated bilateral lower-limb oedema. Given the combination of oedema, chest discomfort, and suspected pulmonary oedema, the initial working diagnosis was acute decompensated heart failure. Intravenous furosemide was initially commenced for presumed fluid overload. Following limited clinical response, a glyceryl trinitrate (GTN) infusion was subsequently commenced.

Bedside transthoracic echocardiography demonstrated significant left ventricular hypertrophy, a small pericardial effusion, and preserved left ventricular systolic function. Although these findings indicated cardiovascular involvement, the preserved systolic function and subsequent clinical deterioration prompted reconsideration of the initial diagnosis. Initial laboratory investigations demonstrated a troponin level of 14.9 ng/L, mild hyponatraemia (sodium 127 mmol/L), a stable estimated glomerular filtration rate (eGFR) of 89 mL/min/1.73 m², creatinine of 59 µmol/L, and a C-reactive protein (CRP) level of <5 mg/L. Serial thyroid function test results throughout the admission and follow-up period are presented in Table 1 and illustrated in Figure 1.

**Table 1:** Summary of thyroid function investigations during admission and follow-up Note: Standard reference ranges are provided in the rightmost column. Directional arrows indicate values outside normal limits relative to the laboratory reference range: (↑) elevated, above the upper limit of normal; (↓) decreased, below the lower limit of normal; (-) within normal limits. Initial presentation demonstrates severe primary hypothyroidism with profound TSH elevation and undetectable free T4, followed by a rapid biochemical response to treatment and complete normalisation at the one-month follow-up. Abbreviation: TSH = Thyroid-Stimulating Hormone.

Investigation	Admission	Day 2	Day 3	Day 6	Discharge Follow-Up (1 Month)	Reference Range
TSH (miU/L)	67 (↑)	43 (↑)	34 (↑)	35 (↑)	0.93 (-)	0.3–5.0
Free T4 (pmol/L)	<2 (↓)	2.0 (↓)	2.7 (↓)	4.4 (↓)	17.6 (-)	9.0–25.0

**Figure 1:**
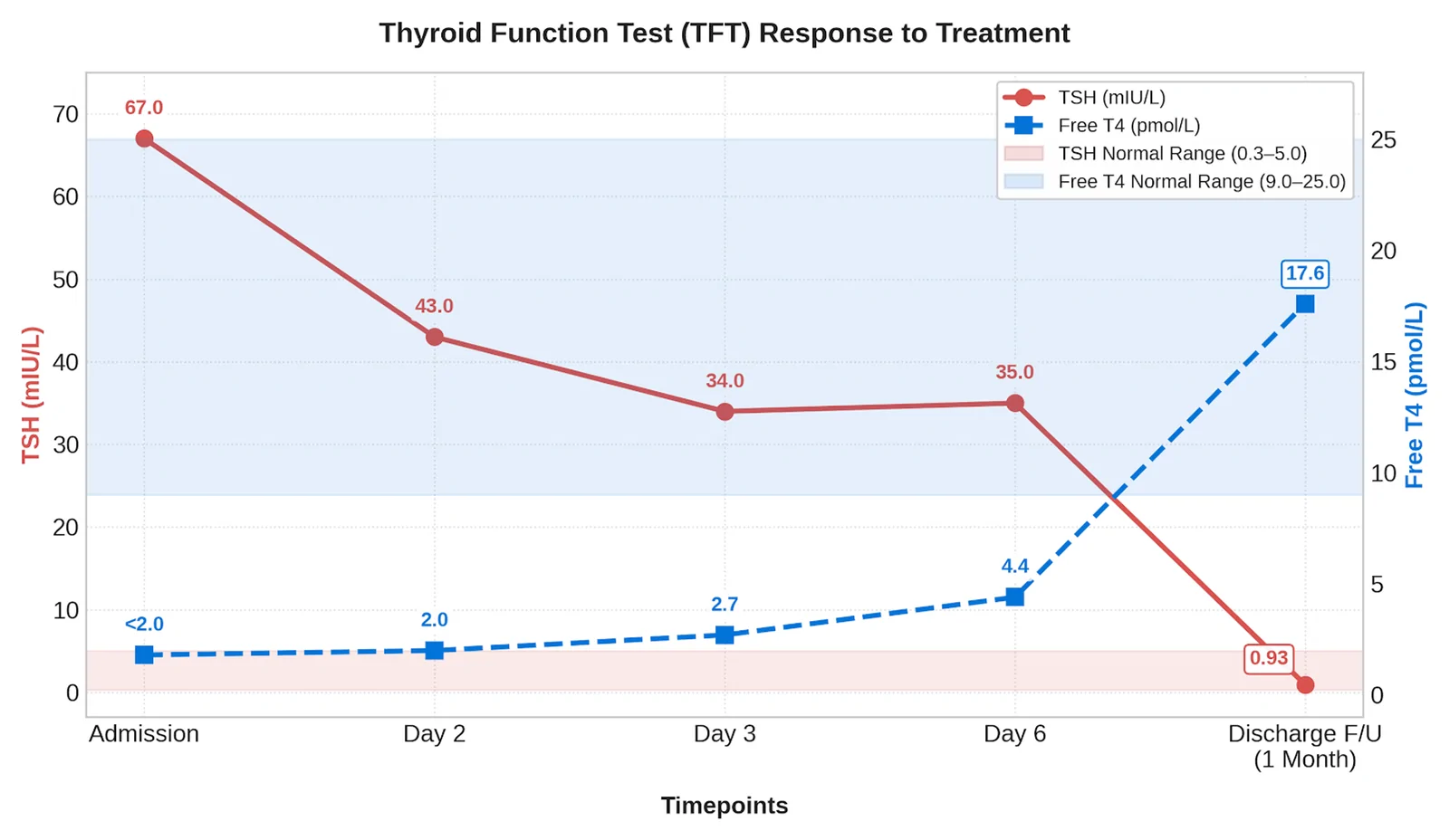
Serial thyroid function tests (TFT) response to treatment across hospitalisation and follow-up Trend of thyroid-stimulating hormone (TSH, red solid line) and free thyroxine (free T4, blue dashed line) levels from admission through a one-month post-discharge follow-up. The light red shaded area denotes the TSH reference range (0.3–5.0 mIU/L). The light blue shaded area denotes the free T4 reference range (9.0–25.0 pmol/L). Initial presentation demonstrates severe primary hypothyroidism with biochemical normalisation achieved by one-month follow-up. Abbreviation: TSH = Thyroid-Stimulating Hormone.

Despite initial treatment, the patient's condition deteriorated over the following hours. She developed worsening hypothermia, hypotension, bradypnoea, and a progressive reduction in consciousness, with her GCS falling to 6/15 (Eye 2, Verbal 1, Motor 3). Arterial blood gas analysis demonstrated respiratory acidosis with hypercapnia (pH 7.263, PaCO₂ 13.30, HCO₃⁻ 18.1)**.** Non-invasive ventilation (NIV) was commenced; however, respiratory failure progressed despite this intervention, necessitating endotracheal intubation and admission to the intensive care unit for invasive mechanical ventilation.

Thyroid function testing demonstrated profound primary hypothyroidism, with a TSH level of 67 mIU/L and a free thyroxine (FT4) level of <2 pmol/L. In the context of severe hypothermia, altered consciousness, cardiovascular instability, and hypercapnic respiratory failure, a diagnosis of myxoedema coma was established. Following discussion with the endocrinology team, she received a stat dose of 75 micrograms of intravenous liothyronine and was commenced on levothyroxine 25 micrograms once daily. This was subsequently increased in 25 microgram increments to a maintenance dose of 125 micrograms once daily.

She was also commenced on intravenous hydrocortisone 100 mg four times daily. As her clinical condition improved during the admission, this was transitioned to oral hydrocortisone at a dose of 20 mg in the morning, 10 mg at lunchtime, and 10 mg in the evening. Hydrocortisone was subsequently discontinued prior to discharge, and she was discharged on levothyroxine 125 micrograms once daily.

Her neurological status improved following initiation of thyroid hormone replacement, and she regained consciousness within 48 hours. She was subsequently successfully extubated; however, persistent hypercapnia required intermittent bilevel positive airway pressure (BiPAP) and non-invasive ventilatory support. Her intensive care admission was complicated by ventilator-associated pneumonia, which responded to antimicrobial therapy. Her respiratory and haemodynamic parameters subsequently improved, with serial thyroid function tests demonstrating progressive biochemical recovery (Figure 1).

She was discharged on long-term levothyroxine replacement (125 micrograms once daily) with planned outpatient endocrinology follow-up. At follow-up one month after discharge, she remained clinically well and reported good adherence to thyroid hormone replacement. Repeat thyroid function testing demonstrated biochemical euthyroidism, with a TSH of 0.93 mIU/L and an FT4 of 17.6 pmol/L (Table 1; Figure 1).

## Discussion

Myxoedema coma carries substantial mortality, with reported mortality rates ranging from 25% to 60% [1-3]. Delayed recognition contributes to poor outcomes, as patients frequently present with non-specific features that overlap with more common acute medical conditions [1-3]. Typical manifestations include hypothermia, altered mental status, bradycardia, hypotension, hypoventilation, and metabolic abnormalities; however, the complete classical presentation may not be present at initial assessment [1-3].

In this case, the patient initially presented with generalised oedema, chest discomfort, and features suggestive of fluid overload, leading to treatment for presumed acute decompensated heart failure. Progressive hypothermia, hypercapnic respiratory failure, hypotension, and deterioration in consciousness subsequently prompted reconsideration of the diagnosis. Retrospective review identified preceding symptoms consistent with chronic hypothyroidism, including fatigue, dry skin, hair thinning, and reduced mobility, although thyroid dysfunction had not previously been investigated. Previous case reports have described myxoedema coma presenting predominantly with respiratory failure or initially being attributed to cardiac, infectious, or respiratory disease [12-14]. Similar diagnostic delays have been reported, with the underlying endocrine disorder only recognised after clinical deterioration. In contrast, our patient presented with prominent fluid overload and apparent heart failure before developing severe hypothermia and progressive hypercapnic respiratory failure, creating diagnostic overlap with both cardiac and respiratory pathology.

Although hypothyroidism is common, a proportion of cases remain undiagnosed [4-6]. Patients with untreated hypothyroidism may remain clinically stable until physiological stress precipitates severe metabolic decompensation [4]. Dutta et al. reported that a substantial proportion of patients with myxoedema coma had no previous diagnosis of hypothyroidism, highlighting that it may be the first recognised manifestation of severe thyroid hormone deficiency [3]. This is consistent with our patient, whose preceding symptoms had not previously prompted thyroid function testing.

The cardiovascular manifestations of myxoedema coma are complex and result from the systemic effects of thyroid hormone deficiency. Reduced thyroid hormone activity decreases myocardial contractility, heart rate, and cardiac output while increasing systemic vascular resistance [7,8]. Reduced renal perfusion and impaired free-water clearance further contribute to fluid retention and may result in peripheral oedema, pleural effusions, and pericardial effusions [7-9]. Consequently, patients may present with clinical features resembling congestive heart failure despite preserved left ventricular systolic function, as observed in this case. Recognition of this association is important because management directed solely towards cardiac failure may not address the underlying endocrine emergency.

Respiratory dysfunction is another important complication of severe hypothyroidism. Reduced central respiratory drive, impaired ventilatory responses to hypoxia and hypercapnia, respiratory muscle weakness, and upper airway soft tissue changes may contribute to alveolar hypoventilation and type 2 respiratory failure [11-13]. Previous reports have highlighted respiratory failure as a predominant presentation of myxoedema coma and a potential source of diagnostic confusion with primary respiratory disease [12,14]. In our patient, progressive hypercapnia despite non-invasive ventilation necessitated invasive mechanical ventilation, followed by improvement after thyroid hormone replacement. The combination of severe hypothermia and hypercapnic respiratory failure was particularly striking and appears to be less commonly described in previous reports.

A notable feature of this case was the coexistence of profound hypothermia and hypercapnic respiratory failure. Hypercapnia can promote peripheral vasodilatation and increased tissue blood flow through carbon dioxide-mediated vascular effects [17]. In contrast, severe hypothyroidism is associated with reduced metabolic activity, impaired thermogenesis, and increased systemic vascular resistance [7,8,18]. The simultaneous presence of marked hypothermia and hypercapnia in our patient therefore represents an unusual clinical observation. Although the underlying mechanism cannot be established from a single case, the profound reduction in thyroid hormone activity may have contributed to impaired thermoregulation and altered the physiological response to hypercapnia. We use the term “cold hypercapnia” descriptively to characterise this unusual combination rather than to suggest a defined physiological syndrome.

The diagnosis of myxoedema coma is primarily clinical and should not be delayed while awaiting laboratory confirmation. Popoveniuc et al. developed a diagnostic scoring system incorporating thermoregulatory, neurological, cardiovascular, gastrointestinal, and metabolic features to support the recognition of myxoedema coma [15]. A score >60 is strongly suggestive of myxoedema coma, whereas a score <25 makes the diagnosis unlikely. Although this scoring system has not been universally adopted, it may be particularly useful in emergency and intensive care settings, where clinical presentations can be atypical and overlap with other critical illnesses. In retrospect, our patient exhibited several features associated with a high clinical suspicion of myxoedema coma, including severe hypothermia, altered consciousness, bradycardia, hypercapnic respiratory failure, and cardiovascular instability. These clinical features, together with the profound biochemical hypothyroidism, supported the diagnosis and prompted urgent thyroid hormone replacement.

Management of myxoedema coma requires a multidisciplinary approach involving thyroid hormone replacement, intensive supportive care, and treatment of precipitating factors [1-3]. Management principles include ventilatory support when required, correction of metabolic abnormalities, careful fluid management, active rewarming, and empirical glucocorticoid therapy until adrenal insufficiency has been excluded [1-3,16]. The optimal thyroid hormone replacement regimen remains debated; however, both levothyroxine (T4) and liothyronine (T3) have established roles. Levothyroxine provides a more stable and sustained hormonal effect, whereas liothyronine produces a more rapid increase in active thyroid hormone levels but may carry a greater risk of cardiac complications, particularly in elderly patients or those with cardiovascular disease [16]. In this case, combined thyroid hormone replacement was associated with rapid neurological recovery, successful discontinuation of mechanical ventilation, and eventual biochemical euthyroidism.

This case reinforces the importance of considering myxoedema coma in older patients presenting with unexplained hypothermia, altered mental status, hypercapnic respiratory failure, or apparent heart failure. The combination of prominent fluid overload, type 2 respiratory failure, and profound hypothermia created significant diagnostic uncertainty, while the coexistence of hypothermia and hypercapnia represents an unusual observation. Early thyroid function testing and recognition of atypical presentations may reduce diagnostic delays and improve outcomes in this rare but potentially reversible endocrine emergency.

## Conclusions

This case reinforces several important clinical lessons. Myxoedema coma should remain an important differential diagnosis in older patients presenting with unexplained hypothermia, altered mental status, hypercapnic respiratory failure, or apparent heart failure, particularly when accompanied by subtle symptoms of chronic hypothyroidism. Early thyroid function testing, timely endocrine involvement, and prompt initiation of appropriate therapy are essential to reduce diagnostic delays and improve outcomes. Furthermore, this case highlights the unusual coexistence of hypothermia and hypercapnic respiratory failure, demonstrating how profound thyroid hormone deficiency may alter expected physiological responses and contribute to diagnostic uncertainty.
